# From perception to action in public health emergencies: a three-gate integrated framework linking risk perception, information engagement, and protective responses

**DOI:** 10.3389/fpubh.2026.1780596

**Published:** 2026-04-01

**Authors:** Junjie Wei, Haoyang Ren

**Affiliations:** School of Intelligent Engineering, Shandong Management University, Jinan, China

**Keywords:** information engagement, protective action decision model (PADM), protective responses, risk perception, risk information seeking and processing (RISP)

## Abstract

Public health emergencies often show a gap between heightened risk perception and sustained protective behavior. People may seek large amounts of information yet fail to adopt recommended measures, implement them inconsistently, or abandon them as guidance evolves and fatigue accumulates. To explain these patterned breakdowns, this paper develops an integrated framework linking risk perception and affect to information engagement and, in turn, to protective responses. The framework synthesizes the Protective Action Decision Model and the Risk Information Seeking and Processing model to treat information engagement—exposure, seeking or avoidance, verification, and processing mode—as the primary bridge between perception and action. It further distinguishes three behavioral outcomes that are frequently conflated in crisis research: adoption, implementation quality, and persistence. Building on this integration, we propose that, in voluntary and information-rich public health crises, sustained high-quality protective responses are most likely when three enabling conditions are met: a trust gate that supports acceptance of messengers and institutions, an efficacy gate that converts threat into feasible action pathways under real constraints, and an information ecology gate that preserves comprehension and belief accuracy under overload and misinformation. The framework yields testable propositions about when perceived risk produces calibrated seeking, guideline-consistent action, and sustained routines, as well as identifiable breakdown signatures when any gate is substantially weakened. By translating the model into actionable design principles for communication and intervention, this theory provides a parsimonious account of why response efforts succeed or fail and offers a practical agenda for strengthening public health preparedness in future crises.

## 1 Introduction

Public health emergencies repeatedly expose a practical paradox. Perceived threat can rise rapidly, information can become ubiquitous, yet protective behavior remains uneven (1, 2). Even when people adopt recommended measures, they often implement them inconsistently, deviate from guidelines, or discontinue them as the event unfolds (3, 4). These gaps matter because crisis protection is rarely a single decision. Many responses require repeated execution, contextual adaptation, and sustained motivation while evidence, policies, and personal constraints change over time (5).

A central feature of contemporary crises is informational turbulence (6, 7). During outbreaks, guidance is updated as knowledge evolves, competing narratives circulate, and people encounter a high-velocity mix of accurate information alongside rumors and misinformation (8, 9). The World Health Organization, in its Overview of the infodemic, describes this condition as an overabundance of information—including false or misleading content—that can generate confusion, increase risk-taking, erode trust in health authorities, and hinder effective response (2). Under such conditions, simply increasing information supply does not guarantee better decisions (8, 10). Information overload can reduce comprehension and promote heuristic processing, while misinformation can inflate perceived threat yet redirect people toward ineffective or harmful actions (11, 12).

Behavioral and communication sciences have studied key components of this puzzle, but often in parallel rather than as a single mechanism. Work on risk perception highlights how perceived susceptibility, severity, uncertainty, controllability, and personal relevance shape affect and intentions (13–15). Health behavior theories emphasize perceived benefits and barriers, self-efficacy, and cues to action as proximal drivers of preventive behavior (16, 17). Risk communication research shows that trust, credibility, and uncertainty communication strongly condition acceptance of recommendations (18, 19). In a separate stream, scholarship on risk information seeking and processing examines why people seek, avoid, verify, share, and differentially process information under uncertainty and emotional arousal (20, 21).

What remains under-specified is the bridge from perception to action. In crisis settings, risk perception does not translate into behavior in a uniform way because information engagement sits in the middle of the chain (22). People may seek intensively yet fail to adopt, adopt but implement incorrectly, or adopt early but later disengage (23). These outcomes are not only a matter of knowledge. They reflect whether people accept the source of guidance, whether they believe action is feasible and effective, and whether the information environment supports accurate understanding and verification (22, 24).

To address this gap, this paper proposes an integrated framework linking risk perception, information engagement, and protective responses in public health emergencies. The framework synthesizes two established models that are often applied separately (21, 25). The Protective Action Decision Model (PADM) specifies a staged pathway from cues and messages to response decisions, emphasizing how individuals receive, attend to, and interpret information and how they form threat perceptions, protective action perceptions, and stakeholder perceptions (26). The Risk Information Seeking and Processing model (RISP) explains the motivations and cognitive processes that govern information engagement, including perceived information insufficiency, channel beliefs, information-gathering capacity, and systematic versus heuristic processing (27, 28). By combining PADM’s decision architecture with RISP’s motivation and processing mechanisms, the proposed framework treats information engagement as the primary pathway through which perceptions and affect shape action.

A further contribution is to specify enabling conditions that are theoretically consequential for this pathway to succeed in voluntary, information-rich crisis settings. We propose three “gates,” understood as functionally defined enabling conditions, that shape whether risk perception and information engagement translate into robust protective outcomes. The trust gate captures acceptance of the messenger and perceived legitimacy of guidance (29). The efficacy gate captures perceived response efficacy, self-efficacy, and barriers that determine feasibility under real constraints (30). The information ecology gate captures overload, misinformation, and platform dynamics that shape comprehension, belief accuracy, and opportunities for verification (2, 10, 31). These gates are not interchangeable: low trust can block adoption even when actions are feasible, low efficacy can erode implementation quality and persistence even when trust is high, and a degraded information ecology can produce mis-implementation, polarized beliefs, and fatigue even when trust and efficacy are favorable (32). Accordingly, the framework predicts discriminable breakdown signatures that sharpen intervention design and empirical tests. Here, ‘breakdown signature’ refers to observable response patterns across adoption, implementation quality, and persistence, not to psychological states.

In summary, this paper (i) integrates PADM and RISP to formalize information engagement as the bridge between risk perception and protective action, (ii) identifies trust, efficacy, and information ecology as three enabling gates under the scope conditions specified below that generate predictable failure modes, and (iii) derives testable propositions about when risk perception produces calibrated adoption, high-quality implementation, and sustained protective responses.

## 2 Core constructs and boundaries

This section defines the key constructs in the proposed framework and clarifies how they are used in public health emergencies. The goal is to make the mechanism empirically testable and to avoid treating distinct outcomes as if they were the same. In particular, we distinguish adopting a protective action from implementing it correctly and sustaining it over time.

### 2.1 Risk perception as a multidimensional construct

In public health emergencies, risk perception is best treated as a bundle of appraisals rather than a single belief. Five dimensions are especially relevant: perceived susceptibility, perceived severity, uncertainty about the threat, perceived controllability, and personal relevance (33). Different combinations can produce different information and action patterns. For example, high severity coupled with low controllability may elicit avoidance or fatalism, whereas high susceptibility with moderate controllability can motivate calibrated seeking and action (21, 30).

Risk perception is also dynamic. New information, personal experience, and social cues can update any dimension, which then alters subsequent information engagement and protective decisions. Measurement can rely on brief validated scales or scenario-based judgments that separately capture each appraisal dimension (34, 35).

### 2.2 Affective responses: arousal, defensive reactions, and fatigue

Affective responses matter because they shape attention, motivation, and processing (21). Acute arousal, such as worry, fear, or anger, can increase vigilance and the perceived urgency to reduce uncertainty (36). At the same time, intense or prolonged arousal can trigger defensive reactions, including denial, reactance, and deliberate avoidance of information that is experienced as threatening or overwhelming (23, 30).

Over longer crisis periods, many individuals experience risk fatigue. Fatigue can reduce the perceived value of continued vigilance, increase disengagement from guidance, and shift processing toward simple heuristics; this is consistent with WHO’s description of pandemic fatigue as a gradual demotivation to follow recommended protective behaviors over time (4, 10). In the framework, affect is not treated as only an upstream cause or a downstream outcome. Instead, it can function as a mediator that channels risk appraisals into information engagement, and as a moderator that amplifies defensive coping under low efficacy or low trust conditions (37).

### 2.3 Information engagement: exposure, seeking/avoidance, verification, and processing

Public health crises unfold in a high-throughput information environment. We use the term information engagement to cover four components that jointly determine what people know, believe, and act upon (21).

Exposure refers to routine or incidental encounters with information through mass media, digital platforms, and interpersonal networks. Seeking and avoidance capture active engagement choices, including searching for updates, consulting experts, postponing exposure, or disengaging from news. Verification and source choice refer to behaviors used to judge credibility, such as cross-checking across sources, consulting trusted professionals, or evaluating evidence quality. Processing mode describes how information is cognitively handled, ranging from systematic evaluation to heuristic reliance.

These components can move in different directions. Exposure may be high while verification is low, or seeking may coexist with shallow processing under overload (38). The framework therefore treats information engagement as a configuration rather than a single variable (39). It encompasses attention allocation, selective exposure, seeking versus avoidance, verification, and interpretive effort. Emotion-regulation processes may further influence this engagement process by shaping tolerance for threatening information and the likelihood of disengagement over time.

### 2.4 Protective responses: adoption, implementation quality, and persistence

In public health emergencies, protective responses include behaviors such as vaccination, masking and ventilation, testing, isolation, hygiene, and timely care seeking (40). We distinguish three outcome dimensions that are often conflated in prior work.

Adoption refers to initiating the recommended behavior. Implementation quality captures whether the behavior is executed correctly and in ways consistent with guidance, including appropriate timing, frequency, and context. Persistence refers to maintaining the behavior over time and adapting it as circumstances and recommendations change (41, 42).

This decomposition is crucial under infodemic conditions. Misleading content may increase adoption of ineffective actions, degrade quality by promoting incorrect implementation, or reduce persistence by increasing fatigue and cynicism (11, 43). Separating these outcomes helps explain why interventions can raise initial uptake but still fail to deliver durable risk reduction.

### 2.5 Scope conditions and boundary setting

This framework is designed as a mechanism-oriented template for understanding why risk perception does or does not translate into protective action adoption, implementation quality, and persistence during public health emergencies. It is intended to be transferable across emergency contexts, but not context-free. The framework therefore requires explicit scope conditions and boundary assumptions.

Governance mode. The framework applies most directly to contexts in which individuals retain at least some behavioral discretion (e.g., vaccination decisions, voluntary testing, home isolation practices, mask use, information sharing). In strongly mandated or enforcement-driven settings, the pathway remains relevant, but the relative importance of gates can shift. In particular, the trust gate may play a weaker role for initial adoption when compliance is compelled, yet remain highly consequential for implementation integrity and persistence, including whether behaviors are performed correctly rather than nominally.

Information environment. The framework assumes that behavior is shaped within an information environment in which people must interpret signals, guidance, and uncertainty. In information-rich settings, dominant ecology risks often involve overload, inconsistency, and misinformation. In information-poor settings, the same gate remains relevant but the failure mode may shift toward access scarcity, delayed updates, and limited verification pathways. Accordingly, the information ecology gate is defined functionally as whether reliable interpretation and verification are possible at a manageable cognitive and practical cost, rather than being tied exclusively to high-volume media environments.

Cultural and political context. The framework does not assume uniform baseline trust, media systems, or political conditions across countries and communities. Institutional legitimacy, messenger credibility, polarization, and platform governance are treated as contextual parameters that shape gate strength and feedback dynamics.

Behavior type. Protective behaviors differ in friction, repetition, and visibility. One-off versus repeated behaviors, private versus socially visible behaviors, and low-friction versus high-friction behaviors are expected to exhibit different gate sensitivities. Accordingly, transferability in this framework should be understood as structural rather than effect-invariant. The pathway architecture is expected to travel across public health emergency contexts, but the relative weight of each gate, the magnitude of particular breakdown signatures, and the strength of cross-gate coupling are expected to vary with governance mode, information environment, cultural-political conditions, and behavior type.

In short, this framework provides a common mechanism architecture for perception-to-action translation, while explicitly acknowledging that contextual conditions shape which gate becomes dominant and how breakdowns manifest in adoption, implementation quality, or persistence. The propositions and design principles that follow should therefore be interpreted as context-sensitive expectations rather than as invariant effect claims across all public health emergency settings. To make the framework’s “transferable but parameterized” status more explicit, Table 1 summarizes how key scope conditions are expected to shift the relative salience of the three gates and, in turn, the dominant breakdown risks across settings.

**Table 1:** Scope conditions and expected variation in gate salience.

Scope condition	Illustrative variation	Gate expected to be relatively more salient	Dominant breakdown risk likely to increase
Governance mode	Voluntary vs. strongly mandated contexts	Trust for initial adoption in voluntary settings; efficacy and ecology for quality/persistence in mandated settings	Non-adoption vs. superficial uptake/poor implementation
Information environment	Information-rich vs. information-poor settings	Ecology in both, but via overload/inconsistency in the former and scarcity/delayed updates in the latter	Confusion/instability vs. incomplete or delayed action
Cultural/political context	Higher vs. lower legitimacy/polarization	Trust, and often trust × ecology coupling, under uneven legitimacy/polarization	Selective acceptance, contested adoption, unstable persistence
Behavior type	One-off/low-friction vs. repeated/high-friction behaviors	Efficacy and ecology weigh more heavily for repeated/high-friction behaviors	Poor implementation, fatigue, discontinuation

## 3 The integrated framework: PADM × RISP for public health emergencies

**Figure 1:**
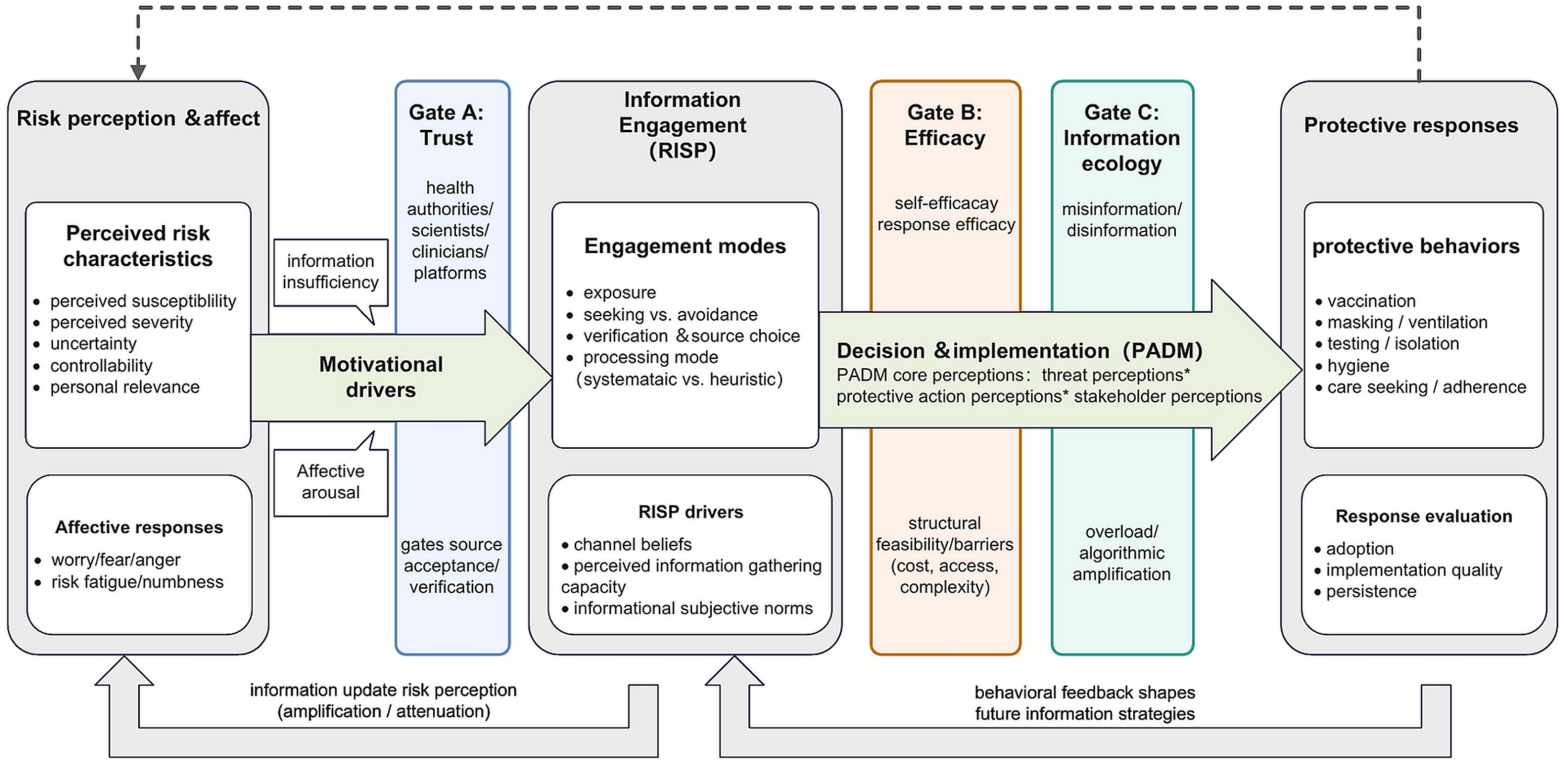
An integrated PADM × RISP framework linking risk perception, information engagement, and protective responses in public health emergencies. Integrated PADM × RISP pathway from risk perception to protective responses, highlighting three enabling gates (trust, efficacy, and information ecology) and dynamic feedback loops. Risk perception and affect motivate information engagement (exposure, seeking/avoidance, verification, and processing), which shape response evaluation and action selection; enactment experiences feed back to update perceptions and subsequent information strategies.

This section specifies how the proposed framework integrates the Protective Action Decision Model (PADM) with the Risk Information Seeking and Processing model (RISP) to explain how risk perceptions translate into information engagement and, ultimately, protective responses. The framework treats information engagement as the mechanism that connects perceptions to action and distinguishes three behavioral outcomes: adoption, implementation quality, and persistence. Figure 1 summarizes the proposed architecture and feedback loops.

### 3.1 PADM as a multi-stage pathway from cues and messages to action

PADM explains protective behavior as a staged process that begins with cues and messages and proceeds through predecisional processes such as exposure, attention, and comprehension. These early stages are consequential because they determine which signals are noticed, which guidance is understood, and which sources are taken seriously (26, 44).

In PADM, predecisional processing feeds into three classes of perceptions that shape response decisions: threat perceptions, protective action perceptions, and stakeholder perceptions. Together, these perceptions guide whether people initiate a recommended action and whether they implement it in ways consistent with guidance (26, 44).

PADM is deliberately general about how information is sought, avoided, verified, and processed under uncertainty (10, 11). In modern crises, patterns of information engagement vary systematically across people and contexts, and they often determine whether messages improve understanding or amplify confusion (11, 38). This is where RISP provides added explanatory leverage.

### 3.2 RISP as a mechanism model of information engagement under risk

RISP specifies why and how people engage with risk information. A central driver of such engagement is perceived information insufficiency—the felt gap between what one knows and what one believes is needed to respond adequately. Information engagement is further shaped by channel beliefs, such as perceived credibility and usefulness of media and interpersonal sources, and by perceived information-gathering capacity, such as skills, time, and access (27, 28, 32, 45, 46).

RISP also distinguishes processing modes. Systematic processing involves effortful evaluation of evidence, whereas heuristic processing relies on cues such as source familiarity, consensus signals, or emotional salience (27, 28, 32, 45, 46). In infodemic conditions, overload and misinformation can shift processing toward heuristics and reduce verification, which can weaken the link between higher concern and better action (2).

### 3.3 Mapping RISP onto PADM: making information engagement the bridge

PADM and RISP are complementary but operate at different analytical levels. PADM provides a decision-process architecture for how people move from cues and messages to protective action decisions, whereas RISP specifies the motivational and cognitive mechanisms through which people seek, avoid, evaluate, and process information under uncertainty. In the present framework, information engagement is treated as the central bridge linking risk appraisal to protective action formation.

The integration follows three principles. First, PADM supplies the staged decision architecture, while RISP specifies the information-related mechanisms that shape whether these stages are populated with reliable, usable beliefs. Second, when PADM and RISP constructs overlap semantically, constructs are retained, merged, or reframed according to their functional role in the pathway rather than preserved as parallel labels. Third, the three gates introduced below—trust, efficacy, and information ecology—are treated as cross-cutting enabling conditions rather than as substitutes for PADM or RISP constructs.

Under this integration, rising risk perception and affect may increase information insufficiency and motivate information engagement, but the downstream effects of engagement depend on source credibility, verification burden, processing mode, and feasibility conditions. Information engagement therefore functions as the mechanism through which risk perception becomes calibrated action, fragmented response, or disengagement. This is the point at which PADM and RISP become mutually reinforcing: PADM clarifies where decisions are formed, while RISP clarifies how information processes determine whether those decisions are formed on usable grounds.

This integration also clarifies why more information does not necessarily improve protective behavior. Depending on trust, efficacy, and information ecology conditions, increased information engagement may support adoption, produce confusion, or intensify avoidance. The framework is designed to explain these divergent trajectories within a single mechanism structure.

Supplementary Appendix 1 provides the construct-level PADM–RISP mapping, indicating which constructs are retained, merged, or reframed and clarifying their role, level of analysis, and temporal position in the integrated pathway. Figure 1 presents the simplified pathway architecture and gate locations.

A further implication of specifying information engagement as the bridge mechanism is that stable individual differences systematically shape how these transitions operate. Although the framework is specified at the individual pathway level, stable individual differences are expected to moderate key transitions. For example, health literacy and numeracy should reduce verification friction and strengthen the conversion from engagement to calibrated beliefs under degraded information environments (47–50). Need for cognition is expected to increase systematic processing and reduce reliance on heuristics under overload (8). Political orientation and identity strength can condition trust-related acceptance of institutional guidance and amplify selective exposure (51). These factors are treated as upstream moderators of gate functioning rather than additional gates.

### 3.4 Dynamic feedback loops in prolonged public health crises

Public health emergencies rarely unfold as single-cycle decision events. They evolve through repeated updates, changing risk conditions, revised guidance, and accumulated behavioral experience (1). To capture this, the integrated framework distinguishes between fast-cycle dynamics and slow-cycle dynamics.

Fast-cycle dynamics refer to short-run processes through which new cues, social signals, or official communications shape risk appraisal, information engagement, and immediate protective responses. These processes typically unfold over hours or days and correspond most closely to the stage-like structure emphasized in PADM (26).

Slow-cycle dynamics refer to changes that accumulate across repeated episodes, including trust erosion or reinforcement, fatigue, shifts in channel use, and changes in the surrounding information ecology. These slower processes alter the baseline conditions under which subsequent fast cycles occur and are especially important in prolonged crises (52).

This distinction helps reconcile PADM’s staged logic with RISP’s recursive information-processing logic (45). In the present framework, a single response episode is the level at which PADM’s staged logic is most visible: appraisal and affect shape information engagement, and information engagement then conditions uptake, implementation quality, and immediate behavioral persistence. RISP contributes the motivational and processing mechanisms operating within this episode, especially perceived information insufficiency, information seeking or avoidance, verification, and the balance between more systematic and more heuristic processing.

Across episodes, however, the model is recursive rather than purely staged. Outcomes from one episode update the baseline conditions for the next by altering trust in institutions and sources, perceived feasibility of action, channel preferences, verification burden, and tolerance for information load. In this sense, PADM primarily describes the within-episode progression of protective decision making, whereas RISP helps explain how repeated information engagement and processing feed back into later episodes. The integration therefore operates through a two-timescale structure: episode-level conversion and between-episode updating. Accordingly, some feedback processes function as rapid bottlenecks within an episode, whereas others operate as slower updating mechanisms that accumulate across repeated episodes.

In a given episode, the pathway may appear stage-like. Across episodes, however, outcomes feed back into both the person and the communication environment. Successful action can reinforce efficacy and trust; repeated inconsistency can increase verification burden; prolonged overload can produce disengagement even when perceived risk remains high (45). The system therefore should not be assumed to move toward a stable equilibrium. In many public health emergencies, it cycles, drifts, or fragments over time. Whether the system moves toward relative stabilization or continues cycling depends on the direction and consistency of these cross-episode updates. Relative stabilization is more likely when guidance remains sufficiently consistent, trusted messengers retain credibility, verification costs stay manageable, and recommended actions are experienced as feasible and worthwhile in everyday life. Continued cycling, drift, or fragmentation is more likely when guidance changes rapidly, competing claims increase verification burden, trust is repeatedly disrupted, or the practical costs of maintaining protection accumulate faster than efficacy can be restored.

The practical implication is that communication effectiveness should be evaluated longitudinally, not only in terms of short-term uptake. A strategy that improves initial adoption may still fail if it degrades trust, increases overload, or weakens persistence over subsequent cycles.

### 3.5 A concrete walk-through: from vaccine risk perception to sustained protective action

To illustrate the integration, consider vaccination decisions in an outbreak. Rising perceived susceptibility and severity can increase information insufficiency and motivate seeking (53). The channels selected and the extent of verification determine whether the individual encounters accurate evidence, misinformation, or conflicting narratives, which in turn shapes protective action perceptions and stakeholder perceptions (54, 55).

If the person accepts the source and believes vaccination is feasible and effective, adoption is likely. Implementation quality depends on understanding eligibility, timing, and contraindications and on the ability to reject misleading claims (56). Persistence and downstream protective routines are shaped by subsequent experiences, updated guidance, and the surrounding information ecology, which can either stabilize calibrated behavior or promote fatigue and discontinuation (2, 57).

## 4 Three gates that explain breakdowns: trust, efficacy, and information ecology

### 4.1 Overview and definition of “gates”

In the integrated PADM × RISP pathway, we define a gate as a functionally defined cluster of enabling conditions that regulates whether momentum is sustained from risk appraisal and information engagement to protective action. We retain the term gate as a heuristic label for recurrent bottlenecks in the perception-to-action pathway rather than as a literal binary switch. Accordingly, the three gates are conceptualized primarily as continuous enabling conditions, not as intrinsically open/closed states. We expect weaker trust, lower efficacy, or a more degraded information ecology to reduce pathway conversion progressively, lowering the likelihood that concern and information engagement will translate into adoption, high-quality implementation, and persistence. Threshold-like disruption may still emerge empirically when degradation becomes sufficiently severe, but such effects are treated here as context-dependent outcomes rather than as the defining structure of the construct. The framework therefore does not assume a universal cutoff at which a gate simply “closes”; where sharper breakdowns appear, they are expected to be behavior-specific, context-dependent, and subject to empirical testing rather than conceptually assumed.

Gates are introduced to foreground a practical question: even when risk concern is present and information is available, what conditions must hold for guidance to be accepted, implemented correctly, and sustained over time? We specify three such conditions—trust, efficacy, and information ecology—because they recur across multiple public health emergency behaviors, occupy distinct bottleneck positions in the pathway, generate diagnostically distinguishable breakdown patterns, and can be directly targeted by public health communication and intervention design. Other factors such as social support, material resources, and cognitive capacity are also important, but in this framework they are treated as contextual influences or as components that operate through these three gates rather than as independent gates in their own right. We reserve gate status for conditions that occupy recurrent bottleneck positions in the perception-to-action pathway, generate relatively distinct breakdown signatures, and are broadly applicable across multiple public health emergency behaviors. In this sense, “gate” is retained as a diagnostic metaphor for functionally privileged conversion conditions, not as a claim that pathway operation depends on a universal binary cutoff. The three gates are distinguished by the proximal function they perform in the pathway rather than by complete empirical independence. The trust gate concerns whether guidance is treated as legitimate, credible, and action-worthy. The efficacy gate concerns whether protective action is seen as feasible, effective, and practically executable under real constraints. The information ecology gate concerns whether information can be interpreted, verified, and stabilized at manageable cognitive and practical cost. Some empirical overlap is expected, especially in prolonged emergencies, but the three gates remain analytically discriminable when they explain different types of pathway failure after mutual adjustment. Empirically, this distinction can be sharpened by varying or modeling one gate while holding the others as constant as possible. Trust-oriented tests would vary messenger legitimacy or institutional credibility while keeping recommendation content broadly comparable; efficacy-oriented tests would vary feasibility supports, barriers, or implementation demands while holding source legitimacy relatively stable; and information-ecology-oriented tests would vary overload, inconsistency, update volatility, or verification friction while holding both source and recommendation content as constant as possible. This strategy does not eliminate overlap, but it clarifies which gate is functioning as the dominant conversion constraint in a given design.

Importantly, the three gates are functionally non-redundant within a given response episode, but they are not causally isolated across time. Within an episode, each gate operates as a parallel bottleneck condition: degradation in any one of them can substantially weaken pathway conversion even when the others remain relatively favorable. Across repeated episodes, however, the gates can become dynamically coupled. A degraded information ecology can erode trust; repeated implementation difficulties can reduce perceived efficacy and increase disengagement; and trust loss can intensify selective exposure, further worsening information ecology. This dual structure—continuous bottlenecks within episodes and dynamic coupling across episodes—helps reconcile gate-based diagnosis with the feedback dynamics observed in prolonged public health emergencies. This functional non-redundancy does not require the gates to be fully independent empirical variables; rather, it requires that each gate identify a different dominant conversion problem in the pathway. Table 2 summarizes the primary bottleneck position of each gate in the integrated pathway and clarifies how each differs from adjacent constructs.

**Table 2:** Distinguishing the trust, efficacy, and information-ecology gates: conceptual boundaries and testable behavioral signatures.

Gate	Primary bottleneck position(s)	Distinct from	Breakdown signature
Trust gate	Primarily conditions the conversion from information engagement to guideline-consistent adoption, while also shaping source selection and message acceptance	Channel beliefs; stakeholder perceptions	High engagement but low or contested guideline-consistent adoption; group divergence (e.g., following outbreak updates closely but rejecting mask or booster guidance from distrusted authorities)
Efficacy gate	Primarily conditions whether accepted guidance can be translated into feasible and correctly implemented action under real-world constraints	Efficacy + barriers	High threat but low action; avoidance/defensive coping; early drop-off (e.g., endorsing home isolation but ending it early because it is not feasible for work or caregiving)
Information ecology gate	Primarily conditions interpretation, verification, and belief stability, while also shaping exposure structure and cognitive burden	Information sufficiency/processing mode vs. ecology quality	Apparent activity or nominal adoption with implementation errors; fatigue and churn (e.g., changing testing advice leading people to follow outdated or conflicting instructions)

### 4.2 The trust gate: legitimacy and acceptance of guidance

The trust gate regulates whether information, recommendations, and warnings are treated as legitimate and action-worthy. In emergency contexts, people often encounter competing claims, evolving evidence, and contested narratives. Under such conditions, acceptance is shaped not only by message content, but also by the perceived credibility, legitimacy, transparency, and fairness of the messengers and institutions behind the message.

Its primary bottleneck position in the integrated pathway lies in the conversion from information engagement to guideline-consistent adoption, but its influence is not limited to that single point. This does not mean that the trust gate encompasses all aspects of source use or information processing. Rather, its distinctive role is to explain whether guidance is granted sufficient legitimacy and action-worthiness to justify uptake, even when exposure, verification, or comprehension have occurred. Trust-related conditions also shape source selection, message acceptance, stakeholder perceptions, and the willingness to continue engaging with official guidance over time. When the trust gate is weakened, information engagement is less likely to convert into adoption, individuals may discount guidance, interpret uncertainty as incompetence or manipulation, or shift toward alternative channels that reinforce prior beliefs. This failure mode can generate a recognizable breakdown signature: high attention or exposure coupled with low guideline-consistent adoption, polarization in perceived credibility, and persistent contestation of official explanations.

### 4.3 The efficacy gate: feasibility and translation from intention to implementable action

The efficacy gate regulates whether accepted guidance can be translated into feasible and correctly implemented action. In public health emergencies, protective behaviors vary widely in friction and constraints: some are one-off (e.g., vaccination), whereas others are repeated and resource dependent (e.g., isolation practices, ventilation routines, testing). Even when perceived risk is high and guidance is trusted, pathway conversion can weaken if people lack self-efficacy, perceive low response efficacy, or face structural barriers that make implementation impractical. Although efficacy judgments can be influenced by the surrounding information environment, the efficacy gate is not defined by clarity or information quality as such. Its distinctive role is to explain whether people judge the recommended behavior to be doable, worthwhile, and sustainable under the material, temporal, and procedural constraints they face.

In this framework, the efficacy gate is closely linked to PADM’s protective action perceptions but is treated functionally as the enabling condition that governs whether acceptance can be translated into feasible implementation under real-world constraints. Key components include response efficacy, self-efficacy, perceived feasibility under constraints (e.g., time, cost, caregiving demands), and perceived barriers. A distinctive breakdown signature of efficacy degradation is that adoption may occur in principle, yet implementation quality is poor or inconsistent, and persistence declines quickly as repeated friction accumulates. In such cases, persuasion alone is unlikely to be sufficient; feasibility supports and barrier reduction become central.

### 4.4 The information ecology gate: interpretability, verification burden, and belief stability

The information ecology gate regulates whether people can form and maintain usable beliefs under the cognitive and social conditions in which information circulates. This gate should not be reduced to the quantity of information alone. Rather, it concerns whether reliable interpretation and verification remain possible at a manageable cognitive cost, given the structure of channels, platform affordances, and the epistemic quality of circulating claims. This gate is not intended to absorb all information-processing constructs from RISP. Rather, it captures the actor-facing informational conditions under which processing occurs, including whether information arrives in manageable volume, whether key messages remain sufficiently consistent across sources, whether verification is practical, and whether the surrounding platform environment stabilizes or fragments belief formation. In this sense, the ecology gate concerns the usability of the information environment, not processing mode in the abstract. Information ecology may also be modeled as a broader contextual moderator at system level. In the present framework, however, it is retained as a gate when operationalized at the actor-facing level because it directly conditions whether information engagement yields interpretable, verifiable, and sufficiently stable action-relevant beliefs.

We conceptualize the information ecology gate as multi-dimensional. Relevant dimensions include: (i) information load and update frequency, (ii) inconsistency and ambiguity across sources, including rapid revisions without explanatory framing, (iii) misinformation exposure and contestation, (iv) verification cost and friction, including the time, skills, and access required to check claims, and (v) platform affordances and channel fragmentation that shape selective exposure and echo dynamics. As this gate degrades, information engagement can become progressively less productive: people may rely more heavily on heuristics, reduce verification, oscillate across sources, or disengage to conserve cognitive resources. The resulting breakdown signature is often high activity but low convergence—unstable beliefs, frequent channel switching, confusion about what is recommended, and reduced persistence despite continuing risk.

### 4.5 Summary: how the three gates jointly produce patterned breakdowns

The three gates are analytically useful because they identify different dominant breakdown mechanisms. In brief, trust primarily conditions legitimacy of uptake, efficacy conditions feasible implementation, and information ecology conditions whether interpretation and verification remain usable under real informational conditions.

Within a single decision episode, the gates function as parallel bottlenecks: strong performance in one gate cannot fully compensate for severe failure in another. High trust does not compensate for infeasible implementation demands; high perceived efficacy does not compensate for deep distrust; high motivation does not compensate for an information environment that makes reliable interpretation prohibitively costly.

Across repeated episodes, however, the gates interact dynamically. Information overload and inconsistency can erode trust; repeated implementation failure can weaken efficacy and promote disengagement; trust loss can intensify selective exposure and worsen ecological fragmentation. The framework therefore supports a diagnostic logic for intervention design: identify whether the dominant breakdown is trust-related, efficacy-related, or ecology-related, and align interventions accordingly—messenger legitimacy and transparency for trust failures, feasibility supports for efficacy failures, and consistency/verification affordances for ecology failures. In doing so, the framework helps explain why public health response can fail not only at the level of adoption, but also at the levels of implementation quality and persistence.

## 5 Core propositions and testable implications

The propositions below specify nine core, distinctive, and falsifiable implications of the integrated PADM × RISP framework with three enabling gates: trust, efficacy, and information ecology. To reduce redundancy and concentrate on the most theoretically diagnostic claims, each proposition is accompanied by a brief scope note, an observable falsification condition, and an indication of whether it concerns primarily fast-cycle dynamics, slow-cycle dynamics, or both. Because the framework is transferable but parameterized rather than context-free, expected effects may vary across boundary conditions. For brevity, some propositions refer to information engagement in summary form, but the construct is treated throughout as a configuration comprising exposure, seeking/avoidance, verification, and processing rather than as a single undifferentiated variable.

P1 [Primarily fast-cycle]. Perceived threat and uncertainty increase information engagement configuration via perceived information insufficiency.

Mechanism: higher threat and uncertainty increase perceived information insufficiency, which in turn increases information engagement configuration. This proposition concerns primarily within-episode movement from concern to information engagement, although repeated episodes may gradually recalibrate what counts as information insufficiency.

Integrated-only novelty: while RISP emphasizes information insufficiency and PADM emphasizes staged decision-making, the integration makes a distinctive prediction about where and why information engagement becomes the bridge from PADM-style threat appraisal into downstream action formation within the same pathway architecture—i.e., insufficiency operates as a tractable transition state linking PADM appraisal stages to RISP engagement processes.

Expected qualitative magnitude: moderate-to-strong for initiating engagement, especially early in crises or during guidance revisions; expected stronger for engagement than for downstream adoption (which is more gate-dependent).

Scope note: This expectation is likely to be strongest in information-rich settings and during periods of guidance revision, when perceived insufficiency is more salient. In information-poor environments, the same mechanism may operate more weakly or be constrained by limited access to updates and verification pathways.

Observable falsification conditions: not supported if (i) information insufficiency does not mediate the threat/uncertainty → engagement association, or (ii) the threat/uncertainty → engagement association remains unchanged after controlling for perceived insufficiency.

P2 [Primarily fast-cycle]. When efficacy is low, high threat increases avoidance/defensive coping rather than effective adoption or persistence.

Mechanism: under high threat, weak efficacy shifts responses toward avoidance, defensive coping, or symbolic action, reducing effective adoption and persistence. This proposition concerns primarily within-episode conversion from concern to action readiness, even though repeated implementation difficulties may later feed back into future efficacy judgments.

Integrated-only novelty: this proposition goes beyond EPPM-like threat–efficacy logic by embedding efficacy as a gate that regulates the conversion of engagement into (i) adoption, (ii) implementation quality, and (iii) persistence within a PADM × RISP pathway. The integrated model predicts the stage-specific breakdown (engagement may rise while adoption/persistence fails) rather than only an overall behavioral outcome.

Expected qualitative magnitude: strong for persistence (drop-off under friction) and moderate-to-strong for adoption of high-friction behaviors; expected weaker for low-friction, one-off behaviors.

Scope note: this expectation should be strongest for repeated, high-friction behaviors and in settings where individuals retain meaningful behavioral discretion. In strongly mandated contexts, low efficacy may be less visible at the level of initial adoption but remain highly consequential for implementation quality and persistence.

Observable falsification conditions: not supported if high threat increases effective adoption and persistence equally across low- vs. high-efficacy conditions, or if efficacy indicators do not predict avoidance/defensive coping after controlling for baseline anxiety/avoidance traits.

P3 [Fast-cycle with possible slow-cycle carryover]. Trust conditions the translation of information engagement configuration into guideline-consistent adoption.

Mechanism: the information engagement configuration is more likely to produce guideline-consistent adoption when trust is high, but more likely to yield contestation, selective acceptance, or non-adoption when trust is low. The immediate moderation predicted here is primarily episodic, but repeated trust failures can accumulate across episodes and alter subsequent willingness to engage with revised guidance.

Integrated-only novelty: PADM includes stakeholder perceptions and RISP includes channel/source beliefs, but neither alone specifies a gate-based conversion prediction: that the information engagement configuration can be intense yet guideline-consistent adoption remain low because trust regulates configuration-to-adoption conversion. The integrated model also ties this to a breakdown signature (high attention with low guideline-consistent adoption) as a diagnostic output.

Expected qualitative magnitude: strong during contested or revised guidance phases; moderate otherwise. Expected stronger for adoption than for implementation quality (which is more efficacy/ecology constrained).

Scope note: the predicted moderation should be strongest in contested, low-consensus, or revised-guidance phases and in contexts where institutional legitimacy and messenger credibility vary substantially across groups. In strongly mandated settings, the trust gate may play a weaker role for nominal uptake but remain important for guideline-consistent implementation and persistence.

Observable falsification conditions: not supported if the association between the information engagement configuration and guideline-consistent adoption does not differ across trust strata, or if the trust moderation effect disappears after controlling for efficacy barriers and ecology indicators.

P4 [Primarily fast-cycle]. Degraded information ecology increases heuristic processing and reduces verification, lowering implementation quality and increasing variability.

Mechanism: when the information ecology gate is degraded (high load, high inconsistency, high contestation, high verification cost), individuals shift toward heuristic processing and reduced verification, producing lower comprehension accuracy and greater variability/errors in how recommended behaviors are implemented. The core claim concerns within-episode shifts in processing and verification under degraded information conditions, although sustained exposure to such conditions may also produce longer-run disengagement.

Integrated-only novelty: RISP addresses processing modes and PADM addresses decision stages, but the integrated gate framework makes a distinct prediction that ecology conditions govern the interpretability/verification bottleneck that specifically degrades implementation quality even when adoption occurs—an outcome distinction not specified by either component model alone.

Expected qualitative magnitude: strong for implementation quality (errors/variance), moderate for adoption delays. Expected larger effects when guidance is complex and frequently updated.

Scope note: this expectation should be most visible in information-rich, high-update, and high-contestation environments, especially when guidance is complex, frequently revised, or difficult to verify. In information-poor settings, ecology-related failure may arise less from overload than from scarcity, delayed updates, and weak verification infrastructure.

Observable falsification conditions: Not supported if ecology degradation indicators are not associated with (i) increased heuristic processing / reduced verification, and (ii) greater implementation variance/error after controlling for education, literacy, and baseline trust.

P5 [Fast-cycle with cumulative slow-cycle implications]. Misinformation can increase behavioral activity while decreasing guideline-consistent adoption and implementation quality.

Mechanism: misinformation can increase apparent activity while redirecting action toward ineffective or harmful alternatives, thereby reducing guideline-consistent adoption and implementation quality. The divergence predicted here may emerge within a single episode, but repeated ecology failures can cumulatively widen the gap between visible activity and guideline-consistent action over time.

Integrated-only novelty: neither PADM nor RISP alone yields the breakdown signature prediction that apparent behavioral activity may rise while guideline-consistent adoption and implementation quality decline. The integrated model links misinformation effects to the ecology gate (verification burden and contestation) and distinguishes misdirected adoption from guideline-consistent adoption.

Expected qualitative magnitude: moderate-to-strong for declines in guideline-consistent adoption and implementation quality, and moderate for increases in misdirected behavioral activity.

Scope note: the predicted divergence between behavioral activity and guideline consistency is likely to be strongest where misinformation is highly salient, verification costs are elevated, and trusted corrective channels are weak or politically contested.

Observable falsification conditions: not supported if misinformation exposure does not predict (i) reductions in guideline-consistent adoption or implementation quality or (ii) greater prevalence of ineffective substitutes, after accounting for baseline distrust and ideology.

P6 [Both fast- and slow-cycle]. Normative and identity alignment shape whether the information engagement configuration translates into guideline-consistent adoption and persistence.

Mechanism: when guidance is norm-congruent for salient groups, the information engagement configuration more strongly predicts guideline-consistent adoption and persistence; when it is identity-incongruent, the same configuration is more likely to produce resistance or selective non-adherence.

Integrated-only novelty: this proposition is anchored in the component theories brought together here rather than added from outside them. PADM highlights the role of social cues, stakeholder responses, and socially mediated protective-action judgments, while RISP implies that information seeking and processing are filtered through prior orientations toward information and sources. The integrated gate framework therefore positions norms and identity alignment as cross-level conditions that modulate gate functioning (especially trust and information ecology) and thus the conversion from the information engagement configuration to guideline-consistent adoption and persistence across multiple outcome dimensions. This is more specific than treating norms as a generic correlate.

Expected qualitative magnitude: moderate-to-strong in polarized contexts and for socially visible behaviors; weak-to-moderate for private, low-identity behaviors.

Scope note: this expectation should be strongest in polarized contexts, under conditions of strong group identity, and for socially visible behaviors. It is expected to be weaker for private, low-identity, or weakly politicized behaviors.

Observable falsification conditions: not supported if identity/norm alignment does not moderate the association between the information engagement configuration and guideline-consistent adoption/persistence, or if any observed moderation is fully absorbed by trust and efficacy measures with no incremental explanatory value.

P7 [Primarily slow-cycle]. Risk fatigue predicts attenuation of the information engagement configuration and reduced persistence over time, even under stable or recurring risk.

Mechanism: over prolonged crises, fatigue predicts attenuation of the information engagement configuration and declining persistence of protective behaviors. This proposition is primarily about across-episode dynamics, because it concerns how repeated burden, disengagement, or fatigue gradually erodes persistence over prolonged emergency periods.

Integrated-only novelty: the integrated model’s explicit fast-cycle vs. slow-cycle architecture predicts that persistence decay can occur even when short-run appraisal remains elevated, because fatigue alters gate baselines (efficacy willingness, trust receptivity, ecology tolerance). This dynamic claim is not entailed by either PADM or RISP in isolation without the integration’s time-scale specification.

Expected qualitative magnitude: strong for persistence in prolonged crises and repeated high-friction behaviors; moderate for attenuation of the information engagement configuration.

Scope note: this expectation is most applicable to prolonged emergencies and repeated protective behaviors that impose ongoing cognitive, practical, or social burden. It should be less pronounced for one-off, low-friction actions unless repeated guidance revisions or sustained uncertainty reintroduce engagement demands.

Observable falsification conditions: not supported if fatigue does not predict within-person declines in engagement or persistence in longitudinal data, or if declines are entirely explained by changes in perceived risk without fatigue effects.

P8 [Both fast- and slow-cycle]. Compound failure of trust and information ecology produces polarized adoption and unstable persistence.

Mechanism: low trust combined with degraded information ecology fragments the information engagement configuration, increases polarization in guideline-consistent adoption, and accelerates discontinuation. The interaction can operate within single episodes when low trust and degraded ecology jointly weaken conversion, but repeated exposure to this combination can also intensify longer-run disengagement, selective exposure, and belief instability.

Integrated-only novelty: the interaction is a distinctive prediction of the three-gate integration: trust and ecology are specified as functionally non-redundant short-run bottlenecks that can become dynamically coupled. The model predicts a specific compound breakdown signature (polarization + instability) beyond what PADM-only or RISP-only would predict from main effects.

Expected qualitative magnitude: strong in high-contestation phases; the interaction is expected to be larger for polarization in guideline-consistent adoption than for mean adoption levels, and larger for persistence instability than for initial uptake.

Scope note: this interaction should be strongest in high-contestation environments characterized by rapid updates, source inconsistency, and uneven institutional legitimacy across audiences. It may be weaker in highly centralized communication systems with strong message discipline and low public contestation.

Observable falsification conditions: not supported if the trust × ecology interaction does not add explanatory power beyond main effects (e.g., non-significant interaction; no improvement in model fit), or if polarization/instability patterns do not differ between low-trust/high-ecology vs. low-trust/low-ecology contexts.

P9 [Both fast- and slow-cycle]. Compound failure of efficacy and information ecology increases implementation errors and accelerates discontinuation.

Mechanism: low efficacy combined with degraded information ecology increases improvised or inconsistent implementation and accelerates discontinuation as cognitive and practical burdens compound. The interaction can undermine implementation within an episode when procedural burden and informational instability coincide, and it can also accumulate across episodes as repeated effort and uncertainty erode persistence.

Integrated-only novelty: this interaction is a signature prediction of the integrated model because it ties a feasibility bottleneck (efficacy gate) to an interpretability/verification bottleneck (ecology gate) and predicts their joint effect specifically on implementation quality and persistence, not merely on adoption.

Expected qualitative magnitude: strong for implementation quality variance/errors and for persistence decay in high-friction behaviors; expected weaker for low-friction one-off behaviors.

Scope note: this interaction should be strongest for repeated, high-friction behaviors that require procedural accuracy under changing or ambiguous guidance. It is expected to be weaker for simple one-off behaviors unless access barriers and verification burdens remain substantial.

Observable falsification conditions: not supported if overload/ecology degradation does not amplify the negative effect of low efficacy on quality/persistence (i.e., no interaction; no differential discontinuation across overload levels conditional on efficacy).

Taken together, these propositions imply that some breakdowns are best understood as acute within-episode conversion failures, whereas others emerge through cumulative across-episode feedback, and many practically important failures involve both temporal scales at once.

## 6 Discussion: design principles and research agenda

This paper proposes a theory-driven synthesis for public health emergencies that links risk perception and affect to information engagement and, in turn, to protective responses. The framework is designed to explain why the same increase in perceived risk can lead to constructive compliance for some people, avoidance or reactance for others, and fatigue-driven disengagement over time. In this section, we translate the framework into design principles for communication and intervention and outline a research agenda that can test the propositions with feasible measures and study designs.

### 6.1 Theoretical contributions and what the framework explains

The framework makes three core contributions. First, it treats information engagement as the central mechanism that connects perceptions to action. Integrating the Protective Action Decision Model with the Risk Information Seeking and Processing model clarifies how motivations, channel beliefs, and processing modes determine the informational inputs that shape response decisions. Second, it distinguishes three outcomes that are often conflated in public health behavior research: adoption, implementation quality, and persistence. This distinction is critical under infodemic conditions because misinformation and overload can increase behavioral activity while reducing guideline-consistent execution and long-run adherence. Third, it specifies three enabling gates—trust, efficacy, and information ecology—that generate structured breakdown signatures. These gates offer a parsimonious explanation for common patterns such as seeking without adoption, high threat with low action, and adoption with poor quality or rapid decay. We derive nine core propositions that predict distinct breakdown signatures from combinations of weakened or degraded gate conditions., enabling sharper diagnosis and more targeted intervention design.

### 6.2 Design principles for public health communication and interventions

The three-gate logic yields a simple design stance. Public health responses are most durable when communication and support jointly open the trust, efficacy, and information ecology gates. The principles below are written as actionable levers that can be implemented by health authorities, healthcare systems, community organizations, and platform partners.

#### 6.2.1 Principles targeting the trust gate

Principle 1: Be transparent about uncertainty and revision while preserving actionability. Communicate what is known, what is uncertain, and what would change the recommendation. When guidance updates, explain the evidentiary reason for the change and the practical implication for behavior. This reduces the risk that revision is interpreted as incompetence or manipulation (58, 59). For example, instead of stating only that guidance may change as evidence evolves, communicators can specify what new evidence or epidemiological change would trigger revision and what people should do differently if that threshold is met.

Principle 2: Separate evidence statements from recommendation statements. Distinguish descriptive claims about the state of evidence from normative choices that reflect feasibility, equity, and resource constraints. This framing helps audiences understand why the same evidence can support different recommendations across settings and can reduce legitimacy disputes (60).

Principle 3: Use credible intermediaries and consistent cross-channel delivery. Trust is often community-specific. Pair official guidance with messengers who are trusted within key audiences and ensure that core messages are consistent across clinical, community, and media channels. Consistency reduces the likelihood that people resolve ambiguity by retreating into identity-consistent information ecosystems (58). For example, a local health department may pair its recommendation with the same message delivered by community physicians, school leaders, or faith-based organizations, so that audiences encounter a consistent core instruction across trusted channels.

Context contingencies for trust-focused design. Trust-oriented principles are especially consequential in voluntary settings, during guidance revision, and in polarized or low-legitimacy environments where acceptance of the messenger cannot be assumed. In strongly mandated settings, these principles may be less decisive for initial nominal uptake, but they remain important for implementation integrity, persistence, and the prevention of performative or purely symbolic compliance.

#### 6.2.2 Principles targeting the efficacy gate

Principle 4: Pair threat information with low-friction, stepwise action pathways. Messages should not only state what to do but also how to do it under common constraints. Checklists, defaults, and implementation prompts can convert intention into correct execution, especially when tasks are repeated (61). For example, instead of advising people simply to “isolate if symptomatic,” guidance can provide a short stepwise prompt such as “test today, stay home until results are known, wear a mask if contact is unavoidable, and seek care if breathing worsens.”

Principle 5: Reduce structural barriers and make feasibility visible. Efficacy is shaped by constraints as much as by beliefs. Policies that improve access, reduce time costs, and provide material support can increase self-efficacy and persistence (62). Communication can reinforce these supports by naming concrete options and troubleshooting barriers.

Principle 6: Design explicitly for persistence. Because many protective behaviors must be sustained, interventions should include renewal cues, feedback on progress, and social reinforcement. Persistence is more likely when people can routinize behavior and when the perceived benefits remain salient without requiring high vigilance (63).

Context contingencies for efficacy-focused design. Efficacy-oriented principles are most critical for repeated, high-friction, and resource-dependent behaviors, where sustained implementation depends on whether action remains doable in everyday life. For one-off or low-friction behaviors, efficacy supports may still matter, but their strongest effects are more likely to appear in implementation quality and persistence than in initial uptake.

#### 6.2.3 Principles targeting the information ecology gate

Principle 7: Reduce cognitive load through stable summaries plus bounded updates. Provide a short, stable “what to do now” summary and place updates in a predictable format. This preserves comprehension under high volume and reduces errors in timing, frequency, and duration (10, 58). For example, during respiratory outbreaks in which isolation, testing, and masking guidance may be revised repeatedly, communicators can retain a stable “what to do now” summary while placing any updates in a clearly bounded and consistently labeled section.

Principle 8: Support verification and inoculate against predictable misinformation. Prebunk common distortions, provide simple verification steps, and highlight cues for source evaluation. When correction is needed, deliver it quickly, clearly, and repeatedly through the channels that affected groups use (64).

Principle 9: Align platform dynamics with public health goals. Work with media and platform partners to limit amplification of falsehoods, increase visibility of authoritative guidance, and add friction to high-risk sharing behaviors (65). Without ecology improvements, increasing message frequency can worsen confusion and fatigue.

Context contingencies for ecology-focused design. Ecology-oriented principles are especially important in information-rich, high-update, and misinformation-prone environments, where overload, inconsistency, and verification burden can undermine calibrated action. In information-poor settings, the same principles may require modification: the priority may shift from reducing overload to improving access, update reliability, and basic verification pathways.

### 6.3 Research agenda: how to test the framework and propositions

The propositions can be tested with designs that match crisis constraints and data availability. First, experiments can manipulate gate-relevant features, such as source credibility and transparency for trust, barrier-reduction cues for efficacy, and curated versus high-volume messaging for ecology (10, 18). In addition, because the gates are conceptualized primarily as continuous enabling conditions, empirical analyses should model gate indicators continuously by default and examine possible nonlinearities using approaches such as spline terms, segmented models, generalized additive models, or mixture-based specifications where context suggests threshold-like breakdowns. Outcomes should include comprehension, verification behavior, adoption, implementation quality, and persistence rather than adoption alone (26). Fast-cycle propositions are especially well suited to event-centered surveys, scenario experiments, message-exposure studies, or experience-sampling designs anchored to discrete communication episodes. Second, longitudinal panel studies can trace dynamics across phases of an emergency to identify fatigue trajectories and feedback from action experiences to later risk perception, trust, and channel choice (66). These designs are especially important for propositions concerning slow-cycle dynamics, such as fatigue, recursive learning, trust erosion, and persistence under prolonged crisis conditions. Propositions that span both temporal scales may require multi-method designs combining event-level measurement with repeated follow-up across phases of the emergency. Third, ecology measurement can combine surveys with behavioral traces, including channel subscriptions, engagement time, sharing behavior, and exposure to misinformation. When temporal dynamics are central, these behavioral traces can also be used to distinguish acute within-episode fluctuations from cumulative across-episode change. Fourth, mixed-method mechanism probing can use interviews or open-ended responses to diagnose why individuals reject guidance, implement incorrectly, or disengage over time (67). These approaches allow direct tests of breakdown signatures and clarify which gate is responsible in a given setting.

To facilitate empirical testing, we provide concise measurement guidance for each gate (Supplementary Appendix 2). In particular, the information ecology gate is operationalized as a multi-dimensional construct capturing volume/cadence, inconsistency, misinformation exposure, verification friction, and platform affordances. Supplementary Appendix 2 also distinguishes subjective, objective/contextual, and behavioral-trace indicators, and notes recommended controls for identifying gate-specific effects in empirical applications.

### 6.4 Positioning the framework relative to EPPM, COM-B, and social-ecological approaches

The present framework is intended primarily as an augmenting mechanism architecture rather than a replacement taxonomy. Its distinctive contribution is not merely to catalogue determinants of protective behavior, but to specify how risk appraisal is translated into protective action under conditions of uncertainty, contested information, and guidance revision (68). More specifically, the framework adds three clarifications that are less clearly specified in adjacent models. First, it treats information engagement as the bridge between appraisal and action rather than as background exposure alone. Second, it distinguishes trust, efficacy, and information ecology as analytically separable conversion conditions that diagnose different pathway failures. Third, it disaggregates behavioral failure into non-adoption, degraded implementation quality, and weak persistence, thereby making visible different breakdown signatures that may otherwise be collapsed into a single outcome.

Relation to EPPM. EPPM is the closest comparator because it highlights the central importance of threat–efficacy alignment in shaping adaptive versus maladaptive responses (69, 70). The present framework retains that insight but extends it into a broader perception-to-action mechanism for public health emergencies. Its added value lies in explaining why even high threat and high efficacy may fail to produce durable guideline-consistent action when trust is low or when the information ecology is unstable. First, it places information engagement (seeking, avoidance, verification, and processing mode) at the center of the mechanism, rather than treating information exposure as a background condition. Second, it specifies trust and information ecology as distinct enabling conditions that can block or distort action formation even when threat and efficacy are high—for example, when guidance is not accepted as legitimate, or when verification burden and source inconsistency prevent stable interpretation. Third, it decomposes “protective response” into adoption, implementation quality, and persistence, thereby explaining why apparent uptake may coexist with poor execution or rapid drop-off—patterns that are often under-specified in EPPM-style outcome treatments. In this sense, the framework does not reject EPPM’s core logic; it embeds that logic within a wider account of information engagement, legitimacy, and ecology-sensitive breakdown under emergency conditions.

Relation to COM-B and general behavior-change frameworks. COM-B (Capability–Opportunity–Motivation) provides one of the most influential general organizing frameworks for behavior change and is highly useful for intervention planning in public health (71). The present framework is compatible with COM-B, but contributes a different kind of explanatory value: a crisis-specific mechanism account of how action fails or succeeds under uncertainty, contested authority, and unstable information conditions. COM-B offers a broad categorization of determinants, whereas the present model specifies a crisis communication mechanism pathway under conditions of uncertainty, rapid guidance revision, and contested information environments. In this context, the gate structure provides diagnostic leverage: it predicts not only whether behavior occurs, but how it fails (non-adoption, low-quality implementation, or weak persistence) and which enabling condition is most likely responsible. In COM-B terms, the efficacy gate corresponds to feasibility-relevant components of capability/opportunity, the trust gate corresponds to legitimacy and acceptance processes shaping motivational conversion, and the information ecology gate corresponds to the epistemic and platform conditions that make information interpretable at manageable cognitive cost. The added value is therefore not a replacement taxonomy, but a mechanism-based account of conversion bottlenecks, outcome-differentiated failure patterns, and intervention-relevant diagnosis in emergency settings.

Relation to social-ecological approaches. Social-ecological models are essential for situating behavior within multi-level contexts (policy, institutions, community norms, and environments) (72, 73). The present framework is complementary rather than competing. Its primary unit of explanation is the individual pathway from appraisal to engagement to action, while institutional structures, platform affordances, and group dynamics enter as contextual parameters that shape gate strength and pathway transitions. In other words, the framework specifies a micro-level mechanism that can be embedded within social-ecological accounts, and it provides a structured way to connect macro/meso conditions (e.g., institutional trust baselines, platform governance, polarization) to observable behavioral breakdown patterns (74). This is why the framework is best understood as a mechanism layer that can be nested within broader ecological explanations, rather than as a self-sufficient substitute for multi-level analysis.

Table 3 summarizes the distinctive comparative position of the framework: it is not a replacement for established models, but a mechanism-oriented extension designed to explain where perception-to-action conversion breaks down under public health emergency conditions. Taken together, these comparisons clarify the manuscript’s distinctive contribution: a parsimonious, testable, and diagnostically useful mechanism model that (i) integrates staged decision structure with information engagement processes, (ii) specifies three functionally distinct enabling gates that regulate pathway conversion, (iii) differentiates protective outcomes into adoption, implementation quality, and persistence, and (iv) supports context-sensitive and temporally explicit diagnosis of why protective action succeeds, fails, or erodes over time.

**Table 3:** Comparative positioning of the three-gate framework relative to EPPM and COM-B.

Feature	EPPM	COM-B	Three-gate framework
Primary focus	Threat–efficacy alignment	General behavior determinants	Perception-to-action conversion in emergencies
Information engagement	Secondary	Not central as a distinct mechanism	Central bridge between appraisal and action
Trust	Not modeled as a distinct gate	Diffuse across motivation/opportunity	Explicit trust gate
Information environment	Limited treatment	Broad opportunity conditions	Explicit ecology gate
Outcome structure	Adaptive vs. maladaptive response	Whether behavior occurs	Adoption, implementation quality, persistence
Diagnostic value	Threat/efficacy imbalance	Determinant mapping	Gate-specific breakdown diagnosis
Best use	Message response under threat	Broad intervention design	Emergency communication under uncertainty

### 6.5 Limitations and boundary conditions

Several boundary considerations follow from the framework’s structure. First, because public health response is often shaped by policy intensity and enforcement, adoption rates may not cleanly reflect underlying trust or efficacy conditions. In mandated contexts, apparent uptake may coexist with low compliance integrity, making it essential to distinguish adoption from implementation quality and persistence when evaluating intervention success.

Second, the information ecology gate is expected to operate across both information-rich and information-poor settings, but with different failure modes. In high-volume environments, overload and inconsistency can elevate verification burden and undermine belief stability. In low-access environments, scarcity and delayed updates can create reliance on informal channels and limit verification, producing different pathways to the same outcome failures. Empirical tests should therefore measure ecology conditions multi-dimensionally rather than equating ecology with volume alone.

Third, cultural and political heterogeneity implies that baseline institutional trust, media systems, and polarization patterns may change the relative weight of gates and the strength of feedback loops. Comparative studies should therefore treat these conditions as parameters rather than assuming uniform effect sizes. The proposition-specific scope notes and the context contingencies attached to the design principles are intended to make these parameter shifts explicit rather than leaving them as a general background qualification. The framework is intended to support such parameterized comparison by offering a consistent mechanism structure and outcome-differentiated breakdown signatures.

Finally, several inference risks warrant attention. Because perceived risk, information engagement, and behavior may co-evolve, cross-sectional designs may conflate cause and consequence. Longitudinal designs, natural experiments (e.g., guidance revisions), and multilevel models that incorporate policy and platform conditions are especially valuable for testing gate dynamics. Measuring all three outcome dimensions—adoption, implementation quality, and persistence—can reduce misinterpretation of “success” that is driven by nominal uptake alone.

These limitations do not weaken the conceptual value of the framework; rather, they clarify the conditions under which it is expected to be most informative and the design features that are most likely to yield decisive tests.

## 7 Conclusion

Public health emergencies demand coordinated, correct, and sustained protective action. The proposed framework explains why the perception-to-action pathway becomes fragile when trust is contested, efficacy is undermined by constraints, or information ecology is degraded by overload and misinformation. By integrating decision architecture with information-seeking and processing mechanisms and by specifying three enabling gates, the framework offers a parsimonious account of patterned breakdowns and a practical guide for designing interventions that improve adoption, implementation quality, and persistence.

## Data Availability

The original contributions presented in the study are included in the article/Supplementary material, further inquiries can be directed to the corresponding author.
