# Supplementary material for: From perception to action in public health emergencies: a three-gate integrated framework linking risk perception, information engagement, and protective responses
Source: Front Public Health. 2026 Apr 1;14:1780596. doi: 10.3389/fpubh.2026.1780596 (PMC13079650; doi:10.3389/fpubh.2026.1780596)
Supplement: Supplementary file 1 [file table_1.docx]

**Supplementary Appendix 1. Construct-level mapping of PADM and RISP in the integrated framework**

This appendix provides a construct-level mapping of the Protective Action Decision Model (PADM) and the Risk Information Seeking and Processing model (RISP) in the integrated framework developed in this article. It identifies which constructs are retained, merged, or reframed, and clarifies each construct’s role, primary level of analysis, and temporal position in the perception–information–action pathway. The purpose of this appendix is to make the theoretical integration explicit, reduce conceptual redundancy, and support interpretability of the main framework.

**Table S1. Construct-level mapping of PADM and RISP in the integrated framework**

| **Source model** | **Original construct** | **Status in this paper** | **Reframed role in integrated pathway** | **Primary level of analysis** | **Temporal position** |
| --- | --- | --- | --- | --- | --- |
| PADM | Environmental/social cues and warnings | Retained | Upstream triggers that initiate risk appraisal and perceived information insufficiency; shape attention and salience | Individual exposure embedded in social context | Fast-cycle input |
| PADM | Threat perceptions (e.g., susceptibility, severity) | Retained | Core risk appraisal state shaping motivation for information engagement and protective action formation | Individual | Fast-cycle appraisal |
| PADM | Protective action perceptions | Retained (clarified) | Evaluation of action usefulness, feasibility, and expected consequences; interacts with efficacy gate as a conversion condition | Individual | Mid-chain decision formation |
| PADM | Stakeholder perceptions | Retained (narrowed) | Perceptions of institutional competence, legitimacy, and messenger trustworthiness; linked to trust gate conditions | Individual perceptions of institutions/actors | Mid-chain; feedback-sensitive |
| PADM | Protective action decision / response | Retained (expanded outcome structure) | Split into adoption, implementation quality, and persistence to distinguish distinct failure modes | Individual behavior (with social consequences) | Outcome stage; feeds back over time |
| RISP | Information insufficiency | Retained | Primary motivational bridge between appraisal and information engagement (felt knowledge gap that motivates seeking/avoidance) | Individual | Fast-cycle trigger; can drift across slow cycles under prolonged crises |
| RISP | Affective responses | Retained | Arousal/concern influencing seeking, avoidance, processing depth, and persistence trajectories (e.g., fatigue) | Individual | Fast-cycle appraisal/motivation; contributes to slow-cycle fatigue |
| RISP | Information seeking | Retained | Component of information engagement (active acquisition of risk-relevant content) | Individual behavior in information environment | Mid-chain processing |
| RISP | Information avoidance | Retained | Component of information engagement; potential breakdown mechanism (defensive disengagement under overload/low efficacy) | Individual behavior | Mid-chain processing |
| RISP | Information processing mode (systematic vs heuristic) | Retained | Mechanism shaping belief quality, verification, interpretability, and susceptibility to misinformation under degraded ecology | Individual cognition | Mid-chain processing |
| RISP | Channel beliefs / source beliefs | Merged (with PADM stakeholder perceptions) | Treated functionally under trust-related pathway conditions (credibility, legitimacy, acceptance of guidance) | Individual perceptions of sources and institutions | Mid-chain + feedback |
| RISP | Perceived information gathering capacity / relevant capacity constructs | Reframed | Primarily conditions information engagement capability (skills, time, access for seeking/verification/processing); secondarily shapes feasibility perceptions relevant to efficacy | Individual (with contextual constraints) | Mid-chain; feedback-sensitive |
| Cross-model integration | Information engagement (umbrella construct) | New integrative construct | Bridge linking appraisal to action via exposure, seeking/avoidance, verification, and processing | Primarily individual, shaped by meso-level ecology | Central pathway connector |
| Cross-model integration | Trust gate | New enabling condition cluster | Regulates acceptance of guidance, messenger legitimacy, and institutional credibility | Cross-level (individual perceptions + institutional context) | Mid-chain + slow feedback |
| Cross-model integration | Efficacy gate | New enabling condition cluster | Regulates conversion of acceptance/intent into feasible, correctly implemented action; affects quality and persistence under friction | Individual + situational constraints | Mid-chain + outcome feedback |
| Cross-model integration | Information ecology gate | New enabling condition cluster | Regulates whether information can be interpreted reliably at manageable cognitive cost (overload, inconsistency, verification friction, platform dynamics) | Cross-level (platform/channel/institutional environment) | Mid-chain + slow feedback |

**Supplementary Appendix 2. Measurement guidance for the three enabling gates**

This appendix provides concise measurement guidance for the three enabling gates in the integrated PADM × RISP framework: trust, efficacy, and information ecology. The purpose is not to prescribe a single universal instrument, but to clarify how the gates may be operationalized in ways consistent with the framework. Across all three gates, indicators should normally be treated as continuous measures rather than dichotomized into open/closed states. Depending on design and sample size, they may be modeled as latent variables, multidimensional composites, or analytically separate dimensions. Because some empirical overlap is possible, identification should rely on gate-specific indicators, mutual controls, and outcome-specific breakdown signatures rather than on an assumption of complete independence.

**Trust gate**

The trust gate concerns whether guidance is treated as legitimate, credible, fair, and worthy of action. Recommended subjective indicators include trust in public health authorities or relevant intermediaries, perceived transparency, and perceived fairness. Relevant objective/contextual indicators may include prior institutional inconsistency, local trust climate, or changes in the coherence of official guidance. Useful behavioral traces include return visits to official sources, reliance on authoritative channels, or switching away from official guidance after revision. Illustrative item stems may include: “I regard guidance from the relevant public health authorities as credible”; “Even when recommendations are revised, I still view the source as acting transparently and in good faith”; and “I am willing to rely on guidance from these institutions when deciding what protective action to take.” These examples are illustrative only and are not intended as a validated universal scale. Trust should be distinguished from the information ecology gate by controlling overload, inconsistency, and verification burden, and from the efficacy gate by controlling feasibility beliefs and implementation barriers.

**Efficacy gate**

The efficacy gate concerns whether recommended action is perceived as feasible, effective, and sustainable under real-world constraints. Recommended subjective indicators include response efficacy, self-efficacy, perceived feasibility, anticipated effort, and perceived procedural clarity. Relevant objective/contextual indicators may include time or cost burden, service access, supply availability, and the stability of implementation requirements. Useful behavioral traces include delayed initiation, incomplete execution, repeated failed attempts, or rapid drop-off after initial uptake. Illustrative item stems may include: “I am confident that I can carry out this recommendation correctly in my daily circumstances”; “Following this recommendation would be effective enough to justify the effort required”; and “Practical barriers make it difficult for me to sustain this behavior over time.” These examples are intended only to clarify operationalization and should be adapted to the behavior and emergency context under study. Where possible, self-efficacy, response efficacy, and implementation barriers should be modeled separately. Efficacy should be distinguished from the trust gate by controlling legitimacy and credibility, and from the information ecology gate by controlling overload, misinformation exposure, and verification burden.

**Information ecology gate**

The information ecology gate should be treated as a multidimensional construct rather than a single undifferentiated score. Relevant dimensions include: (i) information load and update cadence, (ii) inconsistency or ambiguity across sources, (iii) misinformation exposure and contestation, (iv) verification friction, and (v) platform affordances or fragmentation. Recommended subjective indicators include perceived overload, confusion, inconsistency, difficulty checking claims, and instability of guidance. Relevant objective/contextual indicators may include message volume, update frequency, cross-source discrepancy, exposure to misinformation, fact-check availability, and platform amplification conditions. Useful behavioral traces include channel switching, repeated cross-source checking, time spent on verification, sharing of unverified claims, and discontinuous engagement following guidance revisions. Because these dimensions need not move together, multidimensional modeling is preferable unless theory or sample constraints clearly justify simplification. Illustrative item stems may include: “I encountered too much conflicting information to tell what was actually recommended”; “Checking whether claims were accurate required more time or effort than I could realistically manage”; and “Across the sources I use, guidance was stable enough to support confident action.” These examples are illustrative only and are intended to show how overload, verification friction, and belief stability may be captured at the actor-facing level. The information ecology gate should be distinguished from the trust gate by holding messenger legitimacy as constant as possible, and from the efficacy gate by controlling feasibility beliefs and implementation barriers.

**Outcome alignment and controls**

Gate measures should be linked, where possible, to the framework’s three outcome dimensions: adoption, implementation quality, and persistence. Different gates may predict different breakdown patterns across these outcomes. To identify gate-specific effects, studies should normally adjust for key background variables such as age, gender, education, health literacy, numeracy, prior behavior, prior emergency experience, baseline risk perception, and general media-use intensity; depending on context, additional controls may include policy stringency, local threat level, prior institutional trust climate, access to relevant services, and political orientation or identity strength where relevant. Researchers are encouraged to match design choice to temporal logic: event-centered surveys, experiments, and experience-sampling designs are useful for fast-cycle propositions, whereas longitudinal panels, diary methods, repeated follow-up, and cumulative behavioral traces are better suited to slow-cycle dynamics. Where theory suggests threshold-like breakdowns, nonlinear specifications may be tested, but gate indicators should be modeled continuously by default.

**Table S2. Suggested operationalization of the three enabling gates**

| Gate | Core dimensions | Subjective indicators | Objective/contextual indicators | Behavioral traces |
| --- | --- | --- | --- | --- |
| Trust | Legitimacy, credibility, fairness | Trust in authorities/intermediaries; transparency; fairness | Messaging consistency; prior institutional performance; local trust climate | Return visits to official sources; switching away from official channels |
| Efficacy | Response efficacy, self-efficacy, feasibility | Perceived effectiveness; confidence; barriers; anticipated effort | Time/cost burden; service access; supply availability; procedural stability | Delayed initiation; incomplete execution; failed attempts; rapid drop-off |
| Information ecology | Load/cadence, inconsistency, misinformation, verification friction, platform conditions | Overload; confusion; inconsistency; difficulty checking claims | Message volume; update frequency; source discrepancy; misinformation exposure; fact-check availability | Channel switching; cross-source checking; verification time; sharing of unverified claims |
